# *Ficus platyphylla *promotes fertility in female Rattus norvegicus Wistar strain: a preliminary study

**DOI:** 10.1186/1477-7827-9-145

**Published:** 2011-11-02

**Authors:** Chinenye J Ugwah-Oguejiofor, Shaibu O Bello, Raymond U Okolo, Emmanuel U Etuk, Michael O Ugwah, Vincent U Igbokwe

**Affiliations:** 1 Department of Pharmacology, Faculty of Pharmaceutical Sciences, Usmanu Danfodiyo University Sokoto, Nigeria; 2 Department of Anatomy, College of health Sciences, Usmanu Danfodiyo University Sokoto, Nigeria; 3 Department of Pharmacology, College of health Sciences, Usmanu Danfodiyo University Sokoto, Nigeria; 4 Department of Pharmacy, Usmanu Danfodiyo University Teaching hospital, Sokoto, Nigeria; 5 Department of Physiology, College of health Sciences, Usmanu Danfodiyo University Sokoto, Nigeria

## Abstract

**Background:**

*Ficus platyphylla *Delile (family- Moracea) commonly called gutta percha tree is a deciduous plant found in savannah areas. It grows widely in the Northern part of Nigeria, up to 60 ft. high and is known as 'gamji' by the Hausas. The seeds, bark and leaves have been used traditionally in combination to promote fertility. Scientifically, the plant has been shown to have analgesic, anti-inflammatory and CNS effects. The present study was to validate the use of this plant to promote fertility in female Rattus norvegicus Wistar strain using various fertility parameters.

**Methods:**

Female Rattus norvegicus Wistar strain weighing between 150-180 g were randomly selected and divided into two major groups. Each group was subdivided into 5 treatment groups of 100, 200, 400 mg/kg BW of aqueous extract of *F. platyphylla *and a control group of 5 ml/kg of distilled water. A positive control of clomiphene citrate was used. Treatment of the first group was discontinued after 15 days prior to mating (pre-mating treatment group), while the other was treated continuously till delivery (continuous treatment group). At the 10^th ^day, females were sacrificed and implantation sites were checked and embryos counted. Upon delivery, litter sizes were determined and the pups weighed and checked for deformities. Other reproductive indices were calculated. Data were analyzed by one-way analysis of variance and students T-test. Proportions were analysed by Chi square. Statistical evaluations were performed using STATS programs and Graphpad prism, and a difference was considered statistically significant at P < 0.05.

**Results:**

There was a significant reduction in the percentage post implantation losses of both the pre-treatment and the continuous treatment groups when compared to their distil water controls. The litter size of the pre-treatment group was similar to the distil water group while at 400 mg/kg, the continuous treatment group showed an increase in the litter size similar to that of the clomiphene group. There were no observed external deformities in the pups.

**Conclusions:**

Administration of aqueous extract of *F. platyphylla *promotes fertility by reducing post implantation loss and by increasing litter size in female Rattus norvegicus Wistar strain.

## Background

Infertility in humans is defined by the failure to achieve pregnancy after 12 months or more of regular unprotected sexual intercourse [1]. It is a reproductive health problem which affects both men and women of reproductive age in all parts of the world [2,3]. In Nigeria, estimates on the prevalence of infertility vary from region to region. In Africa, women bear the burden of infertility more than the men [2,4,5]. Various social implications associated with infertility abound which range from blame for reproductive failures to life threatening interventions [3]. Therefore, it is not unusual to find victims desperately searching for a solution to this malady.

Traditional medicine is of immense value in the health care systems of developing countries. The World Health Organization (WHO) estimates that more than 80% of health care needs in these countries are met through traditional health care practices [6]. This may possibly be because its affordability and accessibility is more than that of the orthodox medicine [7,8]. A lot of herbs have been used traditionally to treat infertility for example, *Lepidium meyenii (maca), Trifolium pretense (red clover), Dioscorea villosa (wild yam) etc*. In the Northern part of Nigeria (Sokoto), *F. platyphylla *(gamji) has been used for this purpose.

*F. platyphylla *Del. Holl (Moraceae) which is commonly referred to as gutta percha tree, is a deciduous plant locally known as "gamji" in Hausa and widely distributed throughout the savannah region of West African coast.

Traditionally, *F. platyphylla *has been used to treat various fertility problems without scientific basis. Attempts have been made to study the plant but not in connection with its fertility promotion [9-11]. Previous studies on the stem bark of the plant revealed that it possesses antinociceptive, anti-inflammatory, and gastrointestinal activities [9,10], it also has some CNS effects [11], in rodents.

In Sokoto, the decoction of the stem bark, leaves and seeds of this plant are used in combination and taken as tea to promote fertility. Currently, it is considered best practice in ethnopharmacology to evaluate ethnomedicines used; i.e in the form it is used in the traditional medical setting [12]. For this reason, this study was conducted on the extract of the leaves, plants and roots (combined) thus mimicking the way it is prepared for fertility purposes in the ethnomedicinal setting.

The present study was designed to examine the fertility promoting effect of aqueous extract of *F. platyphylla *in female Rattus norvegicus Wistar strain of reproductive age through several female reproductive indices and counting of the implanted embryos at various doses of the aqueous extract and comparing it with Clomiphene citrate.

Clomiphene citrate (CC) is a nonsteroidal triphenylethylene derivative that exhibits both oestrogen agonist and antagonist properties [13]. It is a drug of choice for treatment of various forms of infertility and for ovulation induction [13].

## Methods

### Animal

Rattus norvegicus Wistar strain, of both sexes were obtained from Veterinary institute Vom, Jos in separate cages and kept in the animal house of the Department of Pharmacology Usmanu Danfodiyo University Sokoto.

The animals were kept in well constructed cages that allowed freedom of movement for two (2) weeks for acclimatization to the laboratory conditions before commencement of study. Water and standard rat chow were provided *ad libitum *throughout the period of the study.

180 Female rats weighing between 150-180 g were randomly selected and divided into two major groups. The first group was used to assess the effect of pre-treatment of the animal with the extract, before mating (pre-mating group), while the second group was used to assess the effect on continuous treatment of the animals (continuous treatment group) [14]. The study was conducted in accordance with the Organization for Economic Development (OECD) guidelines on good laboratory practice [15].

### Preparation of the plant material

The stem bark, leaves and seeds of *F. platyphylla *(Gamji) were obtained from the surroundings of Usmanu Danfodiyo University teaching hospital Sokoto on the 6^th ^of November 2008. They were identified at the Taxonomy unit of the Department of Botany, Usmanu Danfodiyo University Sokoto. Voucher specimen was deposited in the herbarium with voucher accession number 003.

They were washed with tap water, cut into pieces and air dried for about 10 days to constant weight. The dried material were pulverised mechanically using a grinding machine into a dry powder and weighed. The powder was subjected to soxhlet extraction using distilled water. The filtrates were evaporated in the oven at 50°C. The extract was stored in a freezer from where it was used when required [16].

The clomiphene citrate used was branded Clomid^R ^obtained from Sanofi-aventis, One Onslow Street, Guildford, Surrey GU1 4YS, UK.

### Treatment of animals for the experiment

The female Rattus norvegicus Wistar strain in each of the two major groups were randomly selected and divided into 5 experimental groups (n = 20). The experimental groups consisted of two control groups and 3 treatment groups. The control groups received 5 ml/kg of the vehicle (distilled water) and clomiphene citrate while the treatment groups received 100 mg/kg, 200 mg/kg and 400 mg/kg body weights each of the extract respectively. The ciomiphene treated group served as control for both the pre-mating and the continuous treatment groups. All experimental groups were treated orally by gastric feeding every day for 15 days prior to mating except the clomiphene citrate group which was dosed for 5 days. On 16th day, dosing was discontinued for the pre-mating treatment group while the continuous treatment group was treated continuously till delivery.

### Mating procedure

On the 16^th ^day, all the rats were housed two per male animal in a cage. Vaginal smears were examined every morning for detection of spermatozoa. The day on which spermatozoa was detected in vaginal smear was considered day 1 of pregnancy [17,14].

### Evaluation of animals

All the animals were inspected daily for signs of abortion, illness and prolong duration of pregnancy.

### Caesarean section for implantation sites

On the 10th day of pregnancy corresponding to mid gestation period, half of the females in all the groups were sacrificed by cervical dislocation. The implantation sites were checked and the embryos counted and weighed.

### Reproductive indices

All the remaining pregnant females were allowed to give birth to their offspring. From pregnancy day 19 the animals' cages were inspected for births. As soon as possible after birth the numbers of viable and death newborns were recorded, the pups were weighed and generally inspected for any deformity up to day 7 after birth.

The following reproductive indices were calculated [17]: Mating index defined as number of sperm positive females/number of mated females × 100, Pregnancy index defined as number of pregnant females/number of sperm positive females × 100, Delivery index defined as number of females delivering/number of pregnant females × 100, Birth live index defined as number of live offspring/number of offspring delivered × 100, Post-implantation loss index defined as number of implantation sites - number of live fetuses/number of implantation sites × 100.

### Statistical analysis

Data were tested for normal distribution and analyzed by one-way analysis of variance (ANOVA) and students T-test. Proportions were analysed by Chi square. Statistical evaluations were performed using STATS programs and Graphpad prism, and a difference was considered statistically significant at P < 0.05.

## Results

### Outcome of fertility tests

In the pre-mating treatment group, there was a significant difference in the percentage post implantation loss in all the treatment groups when compared to the distil water control group (Table 1). The 400 mg/kg showed the highest significant decrease in the percentage post implantation loss index than the control. The lower doses 100 mg/kg and 200 mg/kg maintained the implantations more than the control (Table 1).

**Table 1:** Outcome of fertility tests in the pre-mating treatment group

Outcome/Dose(mg/kg)	100	200	400	D. water	clomiphene
Mated females	20	20	20	20	20
Sperm positive females	20	20	20	20	20
Preg. Females	12	12	16	18	5
Mating index(%)	100	100	100	100	100
Preg. Index(%)	60	60	80	90	25
Post implant. Loss index (%)	10^a^	12^a^	-1.30^b^	32.77	-

Preg.: Pregnancy, Implant.: Implantation. D. water = Distilled water. Data analysed by chi square test. ^a, b^significantly different from the negative control at P < 0.05

In the continuous treatment group, all the treatment groups showed significant decrease in the percentage post implantation loss index with the 400 mg/kg showing the highest reduction when compared to the distil water control group (Table 2).

**Table 2:** Outcome of fertility tests in the continuous treatment group

Outcome/Dose(mg/kg)	100	200	400	D. water	clomiphene
Mated females	20	20	20	20	20
Sperm positive females	20	20	20	18	20
Preg. Females	16	18	20^a^	14	5
Mating index(%)	100	100	100	90	100
Preg. Index(%)	80	90	100	70	20
Post implant. Loss index (%)	-27.25^b^	-31.05^b^	-46.6*	29.45	-

D. water = Distilled water. Data analysed by chi square test.*, ^b^significantly different from the D. water group. ^a ^significantly different from the clomiphene group. Values significant at P < 0.05.

The pregnancy index was lowest in the clomiphene treatment group (Tables 1 and 2). The clomiphene citrate group showed no implantation sites.

### Reproductive index

The result of the reproductive index in the pre-mating treatment group showed no significant difference amongst the groups and when compared to the control. However, there was a significant increase in the number of the pups per litter of the rats by the clomiphene citrate group when compared to all the groups (Table 3).

**Table 3:** Reproductive index in the pre-mating treatment group

Reproductive index/ Dose (mg/kg)	100	200	400	D. water	clomiphene
No of (dams) pups	(5) 30	(5) 36	(6) 42	(6) 43	(5) 45
No of pup per litter	6.0 ± 1.66	7.33 ± 4.10	7.00 ± 1.13	7.17 ± 2.12	9.0 ± 0.20*
Pups body wt (g)	5.17 ± 1.38	4.89 ± 1.19	4.82 ± 0.16	4.54 ± 0.90	4.59 ± 0.15
Delivery index (%)	100	100	100	100	100
Birth live index (%)	100	100	100	100	100
No of pups with external malformations	0	0	0	0	0

D. water = Distilled water. No of pups per litter are expressed as mean ± S.D. Weights of pups are expressed as mean ± S.E.M. *Values are significant at P < 0.05

In the continuous treatment group, the 400 mg/kg of the extract showed a significant increase in the number of pups per litter when compared to all the groups except the clomiphene treatment group. The number of pups' body weight of the same group was significantly more than that of the control groups (Table 4).

**Table 4:** Reproductive index in the continuous treatment group

Reproductive index/ Dose (mg/kg)	100	200	400	D. water	clomiphene
No of (Dams) pups	(5) 26	(5) 31	(6) 50	(5) 25	(5) 45
No of pup per litter	5.20 ± 4.62	6.2 ± 3.06	8.33 ± 0.15*	5.00 ± 2.06	9.00 ± 0.20
Pups body weight(g)	5.60 ± 0.50	5.92 ± 0.08	6.26 ± 0.24*	4.83 ± 0.61	4.59 ± 0.15
Delivery index (%)	100	100	100	100	100
Birth live index (%)	100	100	100	100	100
No of pups with external malformations	0	0	0	0	0

D. water = Distilled water. *Values significant at P < 0.05

### Gestation Length

The gestation length was not significantly different from any of the groups and controls. For the 100, 200, 400, dist. water and Clomiphene citrate, the average gestation lengths were 21.22 ± 0.28, 22.08 ± 0.08, 22.3 ± 0.02, 21.82 ± 0.33, and 22.04 ± 0.33 respectively.

### Deformity

On inspection of the pups at birth and daily up to the 7^th ^day of birth, there were no noticeable external deformities in all the treatment groups.

## Discussion

The results of this study showed that the percentage implantation losses in the various doses of *F. platyphylla *in the pre-mating treatment group were less than the control, with the 400 mg/kg showing the least implantation loss index (Table 1). This suggests that the plant may contain constituents which may enhance or maintain pregnancy. Previous phytochemical studies on the plant, by Ugwah-Oguejiofor *et al*., 2011, showed that the plant contains steroids [18]. Various phytosteroids have been shown to promote fertility [14]. However the type of steroid present in *F. platyphylla *has not been evaluated.

The percentages of the *F. platyphylla *pre-mating treatment group that copulated (mating index) were similar in all the groups. The number of pregnant females was significantly more in the treatment groups than in the clomiphene citrate control group. The reason for this difference will need to be explored by further studies. One possible reason is that the plant may have a different mechanism of action although our data is insufficient to support this.

In the continuous treatment group, the fertility test was similar to that of the pre-mating treatment group, however the post implantation loss index was much less than that of the pre-mating group suggesting that during the continuous administration, the extract offered more protection to the embryos.

It has been shown that *F. platyphylla *causes a decrease in the total white blood cell count and lymphocytes which are functions of immune system [18]. It therefore implies that the possible mechanisms through which *F. platyphylla *may act include increased uterine receptivity and altered immune functions [19] even though more studies are needed to ascertain that. It is believed that functional lymphocytic progesterone binding sites are essential for the maintenance of normal pregnancy, and that progesterone-mediated immunosuppression is needed for the maintenance of normal gestation [20].

The pregnancy indices were similar in all groups except for clomiphene citrate group which was very low (Tables 1 and 2). This could be because even though clomiphene citrate (CC) is an ovulation inducer, its restoration of ovulation does not produce a much higher pregnancy rate. This inconsistency between ovulation and pregnancy rates (only 50% of those who ovulate will conceive) may be partly explained by the peripheral anti-estrogenic effects of clomiphene citrate at the level of the endometrium and cervical mucus or by hyper secretion of luteinising hormone [21]. A poor prognosis for conception is indicated if the endometrial thickness on ultrasound scanning does not reach 8 mm at ovulation [21]. Experience has shown that the prevalence of endometrial suppression is one in every 6-7 patients [21].

Again, the litter size of animals treated with clomiphene citrate were higher than the distil water control (table 4) but similar to the 400 mg/kg treated group this could be because CC blocks the negative feedback mechanism which the rising estradiol levels would normally invoke to reduce discharge of Follicular Stimulating Hormone (FSH). The continued flow of FSH encourages multiple follicle development which is relatively common. The risk of multiple gestations is therefore increased and is estimated at 8-13% [22-24].

In the group treated continuously, it is interesting to note that the group dosed with 400 mg/kg body weight showed a significant increase in the litter size compared to the control and the other groups. This could mean that *F. platyphylla *may have a protective effect on the number of resorptions [20]. It is possible that the extract may help to prevent abortion. It may also increase the rate of multiple pregnancies.

In the reproductive index, both the pre-mating treatment group and the continuous treatment group showed no external malformations in their pups at all dose level until at day 7. This again implies that the extract may not be teratogenic however; more studies are needed to conclude this and the mechanism through which this extract act is yet to be verified.

## Conclusions

In conclusion, aqueous extract of the stem bark, leaves and seeds of *F. platyphylla *seems to promote fertility by maintaining uterine integrity and increasing the number of pups in female Rattus norvegicus Wistar strain without any noticeable teratogenic effect.

## Competing interests

The authors declare that they have no competing interests.

## List of Abbreviations

CNS: Central Nervous System; CC: Clomiphene citrate; FSH: Follicle Stimulating Hormone.

## Authors' contributions

CJU conceive of the study, participated in its design, execution, analysis and interpretation of the data, and drafted the manuscript; OSB participated in the design, coordination, analysis and interpretation of the data, and assisted in proof reading the manuscript of the study; RUO assisted in execution of the experiment and revision of the manuscript; EUE participated in the development of the experiment and critically revised the manuscript; MOU assisted in execution of the experiment and revision of the manuscript; VUI participated in the development of the experiment and critically revised the manuscript.

All authors read and approved the final manuscript.
